# Facilitators and barriers to traditional medicine use among cancer patients in Malawi

**DOI:** 10.1371/journal.pone.0223853

**Published:** 2019-10-21

**Authors:** Jacob Hill, Ryan Seguin, Twambilile Phanga, Agness Manda, Maria Chikasema, Satish Gopal, Jennifer S. Smith

**Affiliations:** 1 University of North Carolina, Chapel Hill, North Carolina, United States of America; 2 Malawi Cancer Consortium and Regional Center of Research Excellence for non-Communicable Diseases, Lilongwe, Malawi; 3 University of Malawi College of Medicine, Blantyre, Malawi; African Population and Health Research Center, KENYA

## Abstract

**Background:**

Increasing access to conventional cancer treatment (CT) in low-income countries (LICs) is an important public health initiative to address the global burden of cancer. However, LICs have a high prevalence of use of traditional and complementary medicine (T&CM). It is important to consider the factors that influence a patient’s choice to use T&CM, CT, or both for their cancer treatment.

**Methods:**

We conducted focus groups among adult cancer patients in Lilongwe, Malawi to identify facilitators and barriers of T&CM use. Focus groups were recorded, transcribed, translated, and underwent thematic content analysis.

**Results:**

Cultural norms, T&CM access, T&CM success, and CT failure were all identified as facilitators to T&CM use. CT success and T&CM failure were identified as barriers. Access and norms appear to determine initial treatment selection, while treatment outcomes dictate continued use of T&CM or CT.

**Conclusion:**

This study identified a pragmatic and experience-based treatment selection process that aligns with the social cognitive theory of behavior and assists in comprehending the factors that influence T&CM use among cancer patients in a low resource setting.

## Introduction

Traditional and complementary medicine (T&CM) is an important healthcare component in low income countries (LICs). The prevalence of T&CM use in LICs is estimated to be between 40–71%, and in sub-Saharan Africa is estimated at 58.2% on average in the general population, but prevalence rates vary widely among studies. [1–5] Data on T&CM prevalence in LICs is scarce, particularly among specific demographic groups and disease cohorts. When considering T&CM use among cancer patients in LICs and lower-middle income countries, the estimated median prevalence is 55%, and 50% in Sub-Saharan Africa (SSA). [6]

The high prevalence of T&CM use in LICs has important clinical implications, especially when T&CM and western conventional treatment (CT) are used concurrently. T&CM use has important historical and cultural significance in diverse settings and populations and may provide benefit when used safely and appropriately. However, concerns regarding T&CM use in combination with CT include potential herb/drug interactions [7], lack of T&CM use disclosure [8,9], directly harmful practices (i.e. non-standardized or unsanitary surgical practices, and/or toxic or contaminated herbal products) [10,11], and delayed or abandoned CT treatment. [12] Concerns regarding CT delay and/or abandonment are particularly important for diseases that benefit from early diagnosis and management with CT, such as cancer or HIV. These clinical and safety considerations are all driven by a patient’s choice and timeliness to seek and use T&CM, CT, or both.

In Malawi, T&CM is an informal healthcare resource that is ubiquitous and widely used throughout the country. [13] Despite recent efforts to increase regulation of T&CM by Malawi’s Ministry of Health, collaboration between the formal health sector and traditional healers remains limited. [14] The formal health care system in Malawi provides access to CT at three levels—community health centers, district hospitals, and central hospitals–including both public sector facilities, which are government funded and free of charge, as well as for-profit and not-for-profit private hospitals and clinics. [13] This range of informal and formal healthcare options along with varying degrees of access, cost, and resource availability creates a complex environment for treatment selection. [14]

The regulation of T&CM from a public health standpoint is complicated and an important issue among healthcare policy groups. [4] Although attempts to better understand and regulate T&CM are ongoing, most research focuses on T&CM components, uses, and effectiveness. Information on facilitators and barriers to T&CM use is limited among cancer patients in Malawi. The objective of this study is to explore facilitators and barriers to T&CM use among cancer patients visiting a public healthcare facility in Malawi. This study is intended to support the World Health Organization Traditional Medicine Strategy 2014–2023, which “aims to support Member States in developing proactive policies and implementing action plans that will strengthen the role traditional medicine plays in keeping populations healthy” [4]

## Methods

We conducted 2 focus groups including adult cancer patients who presented to the Kamuzu Central Hospital (KCH) from January-February 2018 to capture information on facilitators and barriers to T&CM use. KCH is a national teaching hospital in the capital of Lilongwe, which serves cancer patients from the northern half of Malawi, with a catchment area of approximately 9 million. This study was approved by the University of North Carolina (UNC) Institutional Review Board and the National Health Science Research Committee of Malawi.

### Sample and location

We selected a purposive sample of cancer patients receiving treatment at the KCH Cancer Clinic. Specifically, we implemented homogenous sampling, with a cancer diagnosis being the homogenous feature required of all participants. During a regularly scheduled treatment visit, patients of the KCH cancer clinic were approached by author AM and asked to participate in one of two focus groups following completion of a quantitative T&CM survey. Participants were approached while waiting for care, receiving chemotherapy, or immediately after completing their cancer visit at KCH. Eligibility requirements included: 1) being at least 18 years old; 2) previous or current diagnosis of cancer; 3) willingness to provide informed consent; and 4) mentally and physically able to participate. Current or past T&CM or CT use were not required, although all participants were receiving CT for cancer at the time of the focus group. Of 16 individuals recruited, 6 participated in focus group 1 (FG1) and 7 in focus group 2 (FG2). All participants gave written informed consent and were provided travel reimbursement. The focus groups were held in a UNC clinical research building on the KCH campus, which is separate from the KCH Cancer Clinic where participants receive cancer treatment. The focus groups were intentionally conducted outside of the hospital setting to encourage freedom of expression.

### Data collection

Authors JH, JSS, SG, and TP developed the focus group discussion guide (S1 File), which was subsequently translated from English to Chichewa. The guide aimed to assess local attitudes towards T&CM and CT by prompting commentary related to the types, frequency, treatment combinations, and determinants of T&CM and CT use for general and cancer-specific treatment. T&CM was defined as any traditional medicines, herbs, rituals, or prayers that are administered by anyone other than medical professionals or someone otherwise unlicensed to provide medical treatment. CT was described as care received at an established healthcare facility and administered by a trained medical professional. Prior to conducting the two focus groups, the guide was pilot tested on two cancer patients in the KCH Cancer Clinic to determine clarity and identify any necessary revisions.

Author TP, a research coordinator for UNC Project-Malawi who is trained in qualitative interviews, led the focus groups. TP had no clinical or research relationship to the respondents prior to conducting the focus groups. Author AM was also present to take field notes to help provide adequate context to the data obtained from participants. Unique identifiers were assigned for each participant in order to connect discussions with participants without revealing identifiable information during analysis. The discussions, conducted in Chichewa, were digitally recorded and subsequently transcribed directly into English. Transcription and translation services were provided by local Malawian medical translators at UNC Project Malawi, who are fluent in both Chichewa and English to ensure accurate data transfer from audio recording to written transcript. Focus group 1 was 1 hour 10 minutes in length, and focus group 2 was 1 hour 29 minutes in length. Following the second focus group, we assessed for data saturation, and determined that additional focus groups were not needed as no new themes had emerged.

### Data analysis

We analyzed the data using thematic content analysis, a form of descriptive analysis that allowed us to understand and describe determinants of T&CM use. Author RS developed deductive codes based on the discussion guide and inductive codes based on salient topics identified during review and coding of the transcripts. RS developed a codebook and coded the transcripts using Dedoose (version 8.0.35), a qualitative analysis software program. When coding was complete, RS developed data matrices which highlighted prominent themes relevant to T&CM barriers and facilitators and enabled in-depth analysis of the topics presented here.

## Results

Among 13 participants, 8 (62%) were male, 8 (62%) resided in rural areas, and median age was 38 years (range 18–61). All were receiving CT for cancer, 11 (85%) reported prior T&CM use, and 10 (77%) had used CT and T&CM concurrently (Table 1).

**Table 1:** Focus group participants demographic information.

Demographic Category	Participants (n = 13)
**Gender**, N (%)	
Male	8 (61.5)
Female	5 (38.5)
**Age**, Mean (SD) Range	38.4 (12.9) 18–61
**Marital Status**, N (%)	
Single	2 (15.4)
Married	5 (38.5)
Divorced	5 (38.5)
Widowed	1 (7.5)
**Cancer Type**, N (%)	
Skin/Kaposi Sarcoma	5 (38.5)
Lymphoma	4 (30.8)
Cervical	2 (15.4)
Breast	1 (7.7)
Unknown	1 (7.7)
**Education**, N (%)^1^	
None	1 (7.7)
Primary	7 (53.8)
Secondary	5 (38.5)
College	0 (0)
**Home Location**, N (%)	
Village	8 (61.5)
City	5 (38.5)
**Distance from Nearest Healthcare Facility**, Mean (SD)^2^	
Kilometers	4.65 (5.32)
Miles	2.89 (3.31)
**Running Water in Home,** N (%)	
Yes	4 (30.8)
No	9 (69.2)
**Electricity in Home,** N (%)	
Yes	1 (7.7)
No	12 (92.3)
**HIV Status,** N (%)	
Positive	9 (69.2)
Negative	4 (30.8)
**Ethnic/Tribal Group,** N (%)	
Chewa	6 (46.2)
Ngoni	3 (23.1)
Lomwe	2 (15.4)
Yao	1 (7.7)
Tumbuka	1 (7.7)
**T&CM Status (Including Prayer),** N (%)^3^	
T&CM User	11 (84.6)
T&CM Non-User	2 (15.4)
**Conventional/T&CM Use (Including Prayer),** N (%)^4^	
Uses Conventional + T&CM	10 (76.9)
Uses Conventional Only	3 (23.1)
**T&CM Status (Excluding Prayer),** N (%)^3^	
T&CM User	10 (76.9)
T&CM Non-User	3 (23.1)
**Conventional/T&CM Use (Excluding Prayer),** N (%)^4^	
Uses Conventional + T&CM	9 (69.2)
Uses Conventional Only	4 (30.8)

1. Highest completed level of education

2. 0.5 used for responses of <1

3. Ever used T&CM specifically for cancer

4. Current use at time of survey

Participants discussed personal T&CM practices and perceptions as well as T&CM norms and beliefs within their respective communities. Discussions were centered on traditional medicines, herbs, and rituals administered by traditional healers. Although conversations emphasized T&CM use, CT use and its place within the context of T&CM was also an important topic. As participants described specific T&CM features and outcomes, major themes emerged related to its use, which we categorized as either facilitators or barriers. Discussions also highlighted salient themes related to CT that were relevant to T&CM use. Overall, most T&CM facilitators were also CT barriers, and T&CM barriers were CT facilitators.

### T&CM facilitators

In both focus groups, participants discussed various factors that promote T&CM use. We stratified their responses into four major themes: 1) Cultural norms, 2) CT failure, 3) T&CM success, and 4) T&CM Access.

#### Cultural norms

Cultural norms were the most frequently discussed T&CM facilitator. We defined cultural norms as the general knowledge, perceptions, and beliefs associated with T&CM. Participants described the common use of T&CM and widespread knowledge of its components and practices throughout their communities. Experience with T&CM was reported to be especially common among older individuals, who often recommend specific treatments for T&CM self-administration or suggest particular traditional healers for the treatment of certain illnesses. These cultural norms surrounding T&CM use in participant communities are important factors in care seeking decisions and primary facilitators of T&CM.

- “In rural areas, three quarters of our parents like to use traditional medicine. There is a difference between the traditional herbs being used and the illnesses they cure. They know their herbs very well.” P3, Focus Group 2
- “Parents encourage it [T&CM] when they know that for that disease the traditional herbs can help. They encourage you and say, ‘You can do this, it helps. You take this herb and mix it with that herb and then you will be cured.’… For example, I had shingles, therefore my parents said, ‘ah, for this we should go to a certain person and that person will help us.” P5, Focus Group 1

In some cases, cultural norms promoted T&CM dependence while discouraging CT. Participants described a common belief that certain illnesses can only be healed by traditional healers and that some forego CT out of a belief that one should rely entirely on T&CM practices.

- “The benefit of going to traditional healers is that certain diseases cannot be treated at the hospital. For example, there is a disease called arthritis, the illness cannot be fully treated at the hospital, but once you visit a traditional healer and he scarifies you, you are healed.” P7, FG2
- “Some people believe in spirits. They do not go to the hospital; they say, ‘We should believe in the spirits’ and just do it there in the village.” P unknown, FG1

Although the effects of cultural norms on treatment selection of T&CM are relevant for all, both male and female participants indicated that women are most likely to adhere to cultural norms via peer influence and, as a result, were more likely to use T&CM. Less access to money and education, and a greater trust in peers were described as potential reasons for this gender-related difference in adherence to cultural norms.

- “As women we like to chat with our friends and we easily believe the things that they tell us… So we do not really like the hospital, we just say that “this is simple, let me just use this.” P1, FG 1

Community advertisements supporting T&CM use are also important T&CM facilitators. These include standard advertising methods such as signs and billboards, as well as marketers who board public transportation to endorse T&CM products.

- “Even in the buses that we board…we hear it. Someone gets on the bus…they advertise tea bags, ‘When you drink these tea bags you will have severe diarrhea and you will vomit a lot and you will urinate frequently: you are completely healed from the cancer. If you have diabetes, it also goes away with the cancer’… Therefore, because we are people who believe things easily we are carried away and say, ‘At least I can try the tea bags.” P1, FG1

#### CT failure

CT failure was another frequently reported T&CM facilitator, and was described as CT’s general inability to treat a disease or its failure to cure or alleviate symptoms. Failure was reported as being experienced both first- and second-hand, as well as hypothetically. Some described CT failure in terms of a belief that CT cannot treat certain illnesses, while others shared personal or peer experiences of CT failure. In many cases, descriptions of CT failure were followed by successful T&CM treatment of the disease.

- “Let us suppose you have gone to the hospital with a child who has a cough; however, after some time you find that the cough is persisting after she has finished the dose of medication. In that case people say, ‘Let us use traditional medicine, for we know the child will get better” P4, FG2

In cases when CT has exhausted its capacity to cure an illness, participants are sometimes encouraged by CT medical staff to seek care elsewhere, a recommendation that is widely interpreted as an endorsement to use T&CM instead.

- “I went to the hospital on several occasions to be helped and in the end they told me in parables that I should look for other options, “kaponde ponde” [go look around]… Since they were speaking in parables I thought they were suggesting that I should go for traditional medicine to be healed.” P3, FG2

In some cases, CT failure prompts a return to T&CM and discontinuation of CT altogether. However, CT failure was also reported to promote concurrent use of T&CM and CT in hopes that using the two simultaneously would expedite healing.

- “They see that when they are getting treatment from the hospital the problem is not ending, so they think that they are wasting time. They think that when they use both, maybe it will go away quickly.” P4, FG1

#### T&CM success

More than half of participants described treatment success attributable to T&CM as a facilitator to its use. Although one participant shared a personal experience of how T&CM success encouraged further use, all other descriptions of T&CM success were based on accounts from family and peers.

- “When they see that someone else was healed, they also believe that “if I drink it [medicine from traditional healer] I will also be healed’… When you hear it from other people that say ‘this person went to this prophet who prayed for the person and they were cured of the cancer,’ you also have faith that if I go to that prophet and he prays for me then I will also be cured.” P unknown, FG1

Testimonials of T&CM success predominantly come from parents and reinforce cultural norms by promoting T&CM use and, in some cases, resulting in inhibiting CT use.

- “Parents can see that maybe you are suffering from a disease that they have been cured from by the traditional healer. Therefore, they say, ‘Go to this person and get that same medicine and you will be cured’. That is why they are hesitant to come to a place like this, to the hospital.” *P6*, *FG1*

#### T&CM access

Relatively broader access to T&CM treatment, including availability, proximity, and cost, is another primary facilitator to T&CM use. In addition to widespread availability of T&CM components within communities, which enables self-administration without requiring travel, there is an abundance of traditional healers who offer services that are predominantly more proximal than CT.

- “Malawians are very popular with traditional beliefs… If there is something that is well established, it is traditional medicine… We have so many traditional healers in our communities.” *P3*, *FG2*

Beyond ease of use, proximity to T&CM reduces transportation costs and facilitates T&CM use over all forms of CT, which are generally further from participant homes. Moreover, while some noted that T&CM is more expensive than free CT at government hospitals, others indicated that CT is generally the more expensive option, likely because CT centers most proximal to participant communities are often for-pay clinics and access to free CT requires considerable transportation costs.

- “Some of us stay very far from government hospitals. For example, where I stay we just have a CHAM hospital and for someone to say, ‘I will go to CHAM, a paying hospital, when I do not have any money’, it is a problem. Instead, they decide to go with a chicken to a traditional healer to do some rituals.” P5, FG2

Access also facilitates the use of T&CM as a diagnostic tool that allows patients to quickly and affordably diagnose unfamiliar symptoms, as well as a treatment option for acute illnesses requiring urgent care.

- “Sometimes you go to the traditional healer because you do not know that you have cancer. You just see that you are getting sick so you wonder, ‘What disease is this? Therefore, it is better that I go to the traditional healers, maybe they can assist me because I do not know this disease.” P1, FG1
- “It [T&CM] helps with emergency illnesses that just come unexpectedly. For you to go to the hospital it means the sickness will progress. Some do not have transport, so those things [traditional medicine] help with emergency illnesses.” P5, FG1

### T&CM barriers

Primary barriers to T&CM were: 1) T&CM failure and 2) CT success.

#### T&CM failure

T&CM failure includes unsuccessful T&CM treatment and failure of traditional healers to convey competence and generate trust among users. All participants who reported using both T&CM and CT for cancer treatment used T&CM prior to CT. Failed T&CM treatment results in T&CM being viewed as a risky and problematic option because it leads to delayed CT and disease progression.

- “I think the risk that is there is that you delay treating the illness. For example, at first I did not realize that I had cancer. Because of what happens in the village I just placed my belief in that [T&CM]… I went to the traditional healer and they did a ritual with a winnowing tray… they did scarification, then took some herbal leaves, made me drink that… When I drank it I did not see any change but I saw that the disease was getting worse. Instead of getting cured, the sickness was becoming worse up to the point that I went to the hospital by …it is an ambulance that took me from my home when I had gotten to a point where I couldn’t get up on my own, I couldn’t do anything.” P1, FG1

Some indicated the inability of CT to successfully treat certain illnesses was sometimes due to delayed care and disease progression resulting from failed T&CM.

- “We see that those things [T&CM] delay us because the disease spreads and then at the hospital they will tell you, ‘We have failed, there is nothing we can do.’ They use every method and see that the person is not being healed because it has already spread… It is hard for them to cure you because you have delayed yourself with traditional medicine.” P1, FG1

Beyond T&CM failure to treat, some indicated that traditional healers are incompetent in respect to certain illnesses and therefore incapable of effective diagnosis and treatment. The failure of traditional healers to generate trust is often associated with treatment failure and can lead to a shift in perceptions regarding T&CM effectiveness.

- “I do not trust traditional healers and believe that I cannot be helped by traditional healers or their medicines… You find that he will do his rituals to reverse the disease but nothing is happening. They just do this to earn a living out of it. He is just there to spoil things.” P4, FG2

#### CT success

Successful CT comprises accurate diagnosis and effective treatment and was mainly discussed in regards to CT for cancer. All instances of successful CT occurred subsequent to failed T&CM treatment, and effective CT diagnoses were all compared to the diagnostic shortcomings of T&CM. CT success was considered a barrier to current and future use of T&CM, and the combination of T&CM failure and subsequent CT success was reported to diminish support of T&CM use, creating doubts regarding its effectiveness.

- “If I still believed those things [T&CM] I would have been dead by now. When I moved from [place] and came to the hospital… I met this doctor who took a piece of flesh from my body and I was diagnosed with cancer… Soon after starting treatment all the symptoms started to vanish, the tumor started disappearing. Which means I was delayed by traditional beliefs. It was the time of ignorance for me… We should not dwell much on traditional medicine and beliefs. We need to tell people the truth. I believe that I am done with traditional medicine and I could have been dead by now if I had not started chemotherapy.” P5, FG2

## Discussion

This study is the first to document facilitators and barriers to T&CM use among adult cancer patients in Malawi. Focus group findings outline when, why, and how T&CM and CT were reported to be used among patients in a large cancer treatment setting in the capital city of Lilongwe. Although treatment selection can be complicated in resource-limited settings where T&CM is widely available and CT access and resources are variable, [15] our findings demonstrate a competent and pragmatic approach to treatment selection among cancer patients in Malawi. In light of ongoing global efforts to provide equitable access to high-quality healthcare, effective integration of T&CM and CT may be an important priority for which our findings provide valuable insights, especially in the context of cancer care in resource limited settings.

### Treatment selection

Despite the complex nature of the healthcare sector in Malawi, our participants reported treatment selection that was characterized by a systematic approach to care based on various environmental and personal factors which ultimately engender treatment selection skills and establish future care-seeking intentions and behaviors. Cultural norms and access represented important environmental factors that were primary determinants of initial treatment selection, while participants’ personal experiences with treatment outcomes (T&CM/CT success and failure) dictated subsequent treatment selection. Participant experience with all major themes while navigating Malawi’s healthcare system generated greater competence and confidence in selecting appropriate care options.

### Environmental determinants–cultural norms and access

Of the major T&CM facilitators described by our group, cultural norms was the most frequently discussed. Our findings indicate that cultural norms play a major role in care-seeking patterns within communities, where they strongly favor T&CM use and in some cases discourage CT use. These norms are reinforced through encouragement and support of T&CM use from family members and peers, especially those who have first-hand experience with T&CM. A recent systematic review of T&CM use in SSA similarly found that primary drivers of T&CM use included its alignment with users’ sociocultural, religious, and spiritual values, as well as recommendations from trusted peers, including community elders, family, and friends.[5] The powerful effect of cultural norms on care-seeking behavior has also been highlighted in established behavioral theories. For example, the Social Cognitive Theory suggests that normative beliefs and social support are important determinants of individual behavior. [16,17]

Access was another commonly discussed T&CM facilitator among participants and was described in terms of proximity and affordability. Free government-funded healthcare is available throughout Malawi. However, the majority of community-based centers are privately owned, and nearly a third of all health services in the country are provided by a cost-subsidized network, which nevertheless requires significant out-of-pocket payments by patients. [13,14] Despite a range of free and subsidized services, most indicated that CT was relatively more expensive than T&CM. Although this may be due to private clinics being more proximal to participant communities, these study findings are supported by additional quantitative research which found that out-of-pocket costs for CT were higher than T&CM in rural Malawi. [18] Participants also reported that T&CM access was more affordable than CT due to proximity. The reduced travel time and cost associated with T&CM described among our groups has been noted in another study from the region. [19]

**Fig 1:**
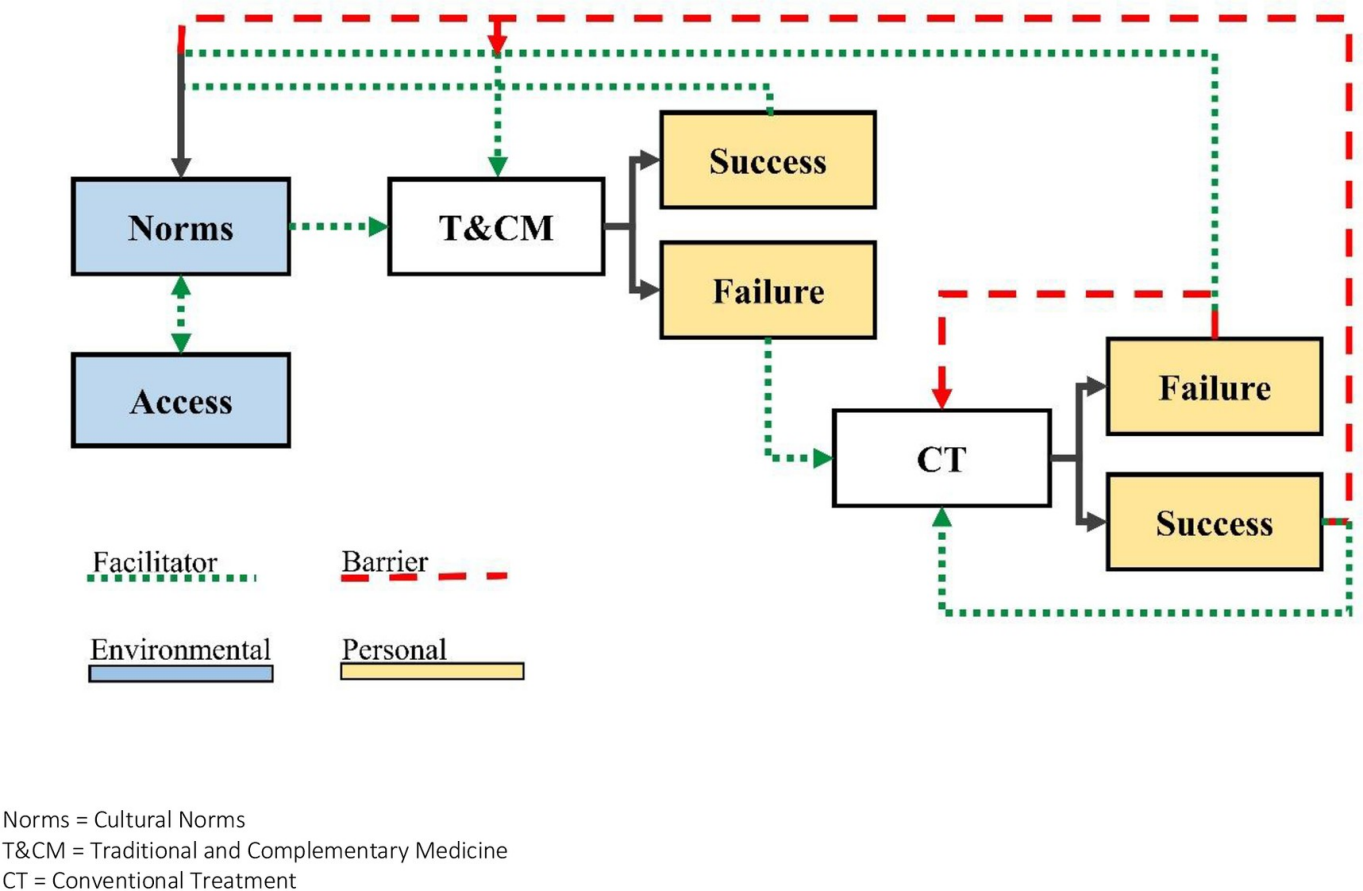
Proposed framework for facilitators and barriers to traditional and complementary medicine use among cancer patients in Malawi. Norms = Cultural Norms T&CM = Traditional and Complementary Medicine CT = Conventional Treatment.

It is likely that these two T&CM facilitators, cultural norms and access, are collaborative in their effect on treatment selection. Longstanding norms generate a high demand for T&CM products and services which in turn drives an increase in supply. As cultural norms increase demand and availability of T&CM, ease-of-access then reinforces cultural norms. Together these factors create an environment that strongly favors T&CM. Moreover, these determinants were important factors in initial treatment selection (Fig 1). Among our group, T&CM was the primary choice for first-line treatment, in line with another study from Malawi. [20] Of participants who discussed using both T&CM and CT for a given illness, all noted that T&CM was their choice of first-line treatment and CT was used only following T&CM failure.

### Personal factors–T&CM/CT success and failure

Following initial treatment selection, continuation of care and future treatment selection is primarily based on personal experience of treatment outcomes. Positive or negative health outcomes following initial treatment was reported to influence participants’ decisions to continue or discontinue use of a given treatment modality, a pattern that has been found in similar settings throughout SSA. [21]

Both T&CM success and CT failure were reported to prompt T&CM use for initial and subsequent treatment. While these treatment outcomes as reported by family and peers reinforce cultural norms and are determinants of initial care selection, participant’s own experiences with T&CM success or CT failure appeared to be paramount in selection of subsequent care.

When participants experience T&CM success, they are more likely to continue using it and tend to recommend its use to others. Additionally, when participants experience CT failure evidenced by no or delayed improvements in health status following CT, most revert to T&CM and discontinue CT use. Other studies from the region have similarly demonstrated that patients often consider persistent symptoms following CT as failed treatment, and subsequently turn to alternative treatment methods. [21] Although the formal health sector in Malawi provides CT in both urban and rural settings, services are often limited as personnel, equipment, and drug resources are scarce throughout the country, [14] and recent findings suggest that limited resources and long wait times at CT facilities reinforce T&CM use and discourage CT. [19]

Conversely, when T&CM fails, patients often turn to CT. T&CM failure includes treatment that renders no improvement in health status or results in negative outcomes, as well as traditional healers’ inability to convey a sense of competence and trust. Although initial treatment selection highly favors T&CM, once participants experience T&CM failure and subsequent CT results are favorable, they are more likely to select CT for subsequent diagnosis and treatment.

These changes in treatment seeking behavior due to treatment outcomes is also supported by the Social Cognitive Theory, which has established outcome expectations as an important cognitive factor in behavior change. [16,17] Our findings, along with other research and theory, suggest that shifts in treatment selection based on the success or failure of T&CM and CT have the potential to reinforce or challenge treatment preferences for certain illnesses (Fig 1).

### Cumulative effect–knowledge and skills

Our findings indicate that exposure to social norms, access to care, and treatment outcomes as individuals navigate the complex healthcare system in Malawi generates behavioral knowledge, skills, and intentions which promote more informed decision making, increase capacity to modify and establish perceptions and preferences for various treatment modalities, and therefore largely determine initial and sustained use of T&CM and CT. Beyond establishing care selection patterns, personal experience with these factors leads to outward support or opposition to certain treatment modalities among peers, with potential implications for care-seeking paradigms within communities. [16]

### Integration of T&CM and CT

The majority of patients in our cohort reported previous T&CM use and described cultural beliefs and access as primary determinants of its use. This is in line with recent analysis by the World Health Organization which indicates that T&CM use throughout SSA is widespread and largely due to access (proximity, availability, and cost), as well as long-standing traditional beliefs and practices. [4] Due to the high prevalence of T&CM use globally and an increasing demand for its services in almost every country, the WHO developed a strategy aimed at integrating T&CM into national healthcare services. [4]

The primary objectives of the WHO Traditional Medicine Strategy include: 1) to establish valid and comprehensive baseline data on which to build national T&CM policies; 2) to strengthen quality assurance, safety, proper use, and effectiveness via regulation of T&CM; and 3) to develop national regulations for T&CM education, training, skills, and services. [4]

Although the most recent WHO global report on T&CM found that many countries in SSA have initiated some form of T&CM policy, country-specific data related to T&CM regulations and programs were unreported for nearly half of the countries in SSA, including Malawi. [13,22] Moreover, Malawi’s most recent health sector strategic plan makes no mention of T&CM other than acknowledging it’s prevalence throughout the country. [13]

With advancing efforts to initiate or expand T&CM policies and programs throughout SSA, our findings may be useful in addressing objective 1 of the WHO Traditional Medicine Strategy by providing a source of relevant baseline data on which to build integrative national healthcare plans that incorporate both CT and T&CM, especially for cancer. For example, as CT likely provides the best pathway to high-quality cancer diagnosis, treatment, and outcomes, improving access to CT at remote healthcare facilities and addressing cultural norms via media campaigns and awareness efforts may help promote CT as a first line treatment for individuals who would otherwise seek T&CM initially. Moreover, improving regulation of T&CM, ensuring adequate personnel and supplies at CT centers, and promoting effective collaboration and referral between T&CM and CT providers can help strengthen the overall network for cancer care. These efforts can provide broad equitable access to effective and integrative healthcare for populations who are likely to continue relying on both T&CM and CT for their healthcare needs.

### Strengths and limitations

All participants in our study were receiving CT for cancer. All were familiar with T&CM practices and most had used T&CM. This is a strength of our study as most patients had experience with both treatment modalities, providing a unique ability to discuss both treatments in detail and outline principal facilitators and barriers to their use. However, our sample may limit generalizability as the determinants of T&CM use among our group may not be applicable to populations lacking experience with or access to CT, especially rural populations who do not ultimately present to central referral hospitals like KCH. Some users of T&CM may have died prior to seeking CT, or have been too sick to access the KCH services. This may introduce selection bias into our study since younger, healthier, patients are more likely to be physically able to access the KCH. This may be reflected by the young median age of participants, 38 years old, which in general is young for an adult cancer population.

## Conclusions

Our findings demonstrate a highly competent, pragmatic, and experience-based treatment selection process among cancer patients in Malawi. Cultural norms and access determine first-line treatment selection, while treatment outcomes determine continuation of care and future treatment selection. Behavioral skills acquired via individual experience with these determinants establishes treatment intentions and perceptions that may create a feedback loop to influence care-seeking norms within communities. Addressing these determinants, and perhaps improving the interaction between parallel T&CM and CT health care systems, should be considered as cancer control programs in similar LIC settings aim to provide care to populations in the most efficient and effective manner under highly resource-constrained conditions. However, additional research is needed in other resource limited settings to confirm the findings of this study.

## Supporting information

S1 File Focus group discussion guide. (DOCX)
