# Supplementary material for: Facilitators and barriers to traditional medicine use among cancer patients in Malawi
Source: PLoS One. 2019 Oct 21;14(10):e0223853. doi: 10.1371/journal.pone.0223853 (PMC6802829; doi:10.1371/journal.pone.0223853)
Supplement: S1 File — (DOCX) [file pone.0223853.s001.docx]

**TCAM Focus Group Discussion Guide**

University of North Carolina, Chapel Hill, USA

UNC-Project Malawi, Lilongwe, Malawi

ENGLISH VERSION

**Study Title**

Concomitant Conventional Treatment and Traditional, Complementary, and Alternative Medicine (TCAM) Use by Cancer Patients in Malawi

**Abbreviations**

TCAM: Traditional, complementary, and alternative medicine

Conventional Cancer Care: Allopathic medical approaches recognized as the standard of care for cancer treatment, often including chemotherapy, immunotherapy, hormone therapy, surgery and/or radiation.

**Contents**

1. Consent Process *(approximately 10 minutes)*
2. Introductions *(approximately 10 minutes)*
3. Assessing local attitudes towards TCAM use and conventional treatment for cancer *(approximately 35 minutes)*

V. Closing *(approximately 5 minutes)*

**Total Time** **of Focus Group Discussion (FGD)**

Approximately 60-90 minutes (including consent process)

**Instructions**

The white text that is highlighted in black is to be followed by the FGD moderator, but not read aloud to participants. It is recommended that the main questions and follow-up questions that are listed on the FGD guide be asked in order. The follow-up questions are intended to guide the discussion, but not all of these questions need to be asked to answer each of the main focus group questions.

Participants may respond to one question in a manner that answers other questions from the FGD guide. It is important for the moderator to be responsive to the group and adjust the order of the questions. If the group has answered a question out of order, it is okay to acknowledge that the group has already shared responses to that question and then ask any follow-up questions to elicit further information.

1. **CONSENT PROCESS** *(approximately 10 minutes)*

Provide two copies of the informed consent form to each participant. Read aloud the consent form to the group for the benefit of participants with limited or no literacy skills. Provide participants an opportunity to ask any questions. Confirm with each participant that they have provided written consent to participate and have no further questions. Have each participant sign both copies of the consent form. Provide each participant with a copy of the consent form, and keep a signed copy to place in the filing cabinet designated for this study.

1. **INTRODUCTION** *(approximately 10 minutes)* TO BE READ BY THE MODERATOR

Thank you for taking the time to join this discussion today. My name is Twambilile Phanga and I am going to lead our discussion today. The purpose of this discussion is to learn about the reasons adults chose to use traditional, complementary, and alternative medicine and/or conventional treatment for their cancer.

This study is being conducted in Malawi by the University of North Carolina in the United States. This research study has been approved by the UNC Institutional Review Board and the Malawi National Health Sciences Research Committee.

Before we start, I would like to remind you that there are no right or wrong answers in this discussion. I am interested in knowing what each of you think. Please be honest and share what you think, even if you don’t agree with others in the group. It is very important that I hear all of your opinions, but you may choose not to answer a question at any time.

Let's start by going around the circle and have each person introduce themselves. I would like each of you to state either a first name or a nickname that you would like to use for the discussion. Your name will not be used when we summarize group responses, or in any written reports.

Allow time for participant introductions.

Thank you. It’s nice to meet all of you!

Establish ground rules.

Write on large pad paper and have visible throughout the group.

Let’s make a list of rules to guide our discussion today. I would like for us, as a group, to agree that what is shared here today stays within our group. In other words, to make sure that everyone feels comfortable sharing their opinions, I want us to agree that we will not talk about what we discuss today with people who were not in this group. We have a few other ground rules to cover, and let me know if you have any others that you would like to add.

**Ground Rules**

- Everything we talk about today is private
- Use first names only when referring to other participants
- There are no right or wrong answers
- It is important that we respect everyone’s opinions, even if they are different from our own
- We want to hear from everyone

Can we as a group agree on these ground rules? Would you like to add any other ground rules? Do you have any questions before we begin recording the discussion?

BEGIN THE RECORDING DEVICE(S)

STATE THE DATE, FOCUS GROUP NUMBER, AND PLACE OF THE FOCUS GROUP DISCUSSION AT THE BEGINNING OF THE RECORDING.

1. **ASSESSING LOCAL ATTITUDES TOWARDS TCAM USE AND CONVENTIONAL TREATMENT FOR CANCER** *(approximately 35 minutes)*

**Participant Instructions:** We will start by asking questions about the community’s attitudes and beliefs about the use of traditional, complementary, and alternative medicine. Traditional, complementary, and alternative medicine may include treatments such as spiritual healing, prayer, herbs, vitamins and minerals, and traditional healing rituals to name a few. These treatments are usually provided by a local traditional healer, and not usually used by a licensed physician/medical doctor. For the rest of this focus group, we will only refer to these treatments as traditional medicine.

1. **What type of traditional medicines and practices are commonly used in your community?**
   1. Follow-up: **Who provides the recommendations for these traditional medicines and practices?**
2. **What are the different reasons people use traditional medicines and practices (example: for a specific illness, to bring good fortune, etc.)?**
3. **What are some of the benefits of using traditional medicine?**
   1. Follow-up: **Do you think there are risks to using traditional medicine? If so, what are they?**

Now I will ask questions specifically about the use of traditional medicines for cancer.

1. **Do you think it is common for people to use traditional medicines specifically for cancer?**
   1. Follow-up**: Why do you think it is common, or uncommon, for people to use these medicines for cancer?**
2. **Is traditional medicine use for cancer more common among men, women, or children?**
   1. Follow-up: **Why do you think it is more common among this group(s)?**
3. **Do people use traditional medicine for specific reasons, such as to cure the cancer, to reduce the symptoms of cancer, or to prevent getting cancer?**
   1. Follow-up**: Why do you think they use traditional medicines for these specific reasons?**
4. **What types of traditional medicines are used in your community to help treat cancer?**
   1. Probe if needed: **For example, do you think spiritual healing, herbs, or vitamins and minerals are the most commonly used traditional medicines for cancer?**
   2. Follow-up: **Why do you think these are the common traditional medicines used for cancer?**

Now I will also ask questions about the community’s attitudes and beliefs about conventional cancer care. Conventional cancer care includes using drugs such as chemotherapy to treat the cancer, and is usually provided by a medical doctor at a hospital. Conventional cancer care can also include surgery and radiation therapy.

1. **Do you think most people use only conventional treatment, only traditional medicine, or both for their cancer?**
   1. Follow-up: **Why do you think people choose to use only conventional treatment, only traditional medicine, or both for their cancer treatment?**
2. **Why do members of the community choose to combine traditional medicines with conventional cancer treatment?**
3. **Do you think there are risks to combining conventional cancer treatment with traditional medicines?**
   1. Follow-up**: If so, what do you think those risks are?**
4. **Is it common for people to see a traditional healer before seeing a physician/medical doctor for their cancer symptoms?**
   1. Follow-up: **Why do people see a traditional healer first before going to a medical doctor for their cancer symptoms?**
5. **Should medical doctors and traditional healers work together to help treat cancer?**
   1. Follow-up: **Why should medical doctors and traditional healers to work together?**
6. **CLOSING** *(approximately 5 minutes)*

Thank you for your thoughts. We are very grateful for what you have shared with us today. Is there anything else that you would like to add to the discussion?

**Thank participants for their time and distribute COMPSENSATION FOR TRAVEL.**
